# A microbe–drug association prediction model based on graph attention networks and rotation forest

**DOI:** 10.3389/fbinf.2026.1871436

**Published:** 2026-08-12

**Authors:** Jing Li, Juncai Li, Qijia Chen, Zhong Wang, Xianzhi Liu, Mingmin Liang, Junzhuang Wang, Hongyuan Ding, Bin Zeng, Lei Wang

**Affiliations:** 1 School of Information Engineering, Hunan Vocational College of Electronic and Technology, Changsha, China; 2 School of Humanities and Education, Hunan Vocational College of Electronic and Technology, Changsha, China; 3 Big Data Innovation and Entrepreneurship Education Center of Hunan Province, Changsha University, Changsha, China

**Keywords:** graph attention network, microbe–drug network, prediction model, rotation forest, similarity measure

## Abstract

**Background:**

In recent years, with the diversification and expansion of drug research in the medical field, the widespread use of drugs, particularly antibiotics, has led to increased microbial resistance. Consequently, exploring potential associations between drugs and microbes has become critically important. However, traditional biological experiments are extremely expensive and time-consuming. Therefore, developing more effective computational models for predicting potential associations between microbes and drugs is both essential and challenging.

**Results:**

We proposed GATROF, a hybrid heterogeneous graph-based framework for microbe–drug association prediction. In GATROF, by integrating multiple microbe–drug–disease similarity measures, we first constructed two distinct microbe–drug networks. In addition, based on different features of microbes and drugs, we further constructed two novel microbe–drug feature matrices. On this basis, the microbe–drug networks and the constructed feature matrices were further used in a Graph Attention Network to learn complementary topology-aware representations of microbes and drugs. These GAT-derived representations were then integrated with the constructed drug-side and microbe-side feature matrices and input into a Rotation Forest classifier for final association prediction. Experimental results and case studies demonstrated that GATROF predicts microbe–drug associations more accurately than existing state-of-the-art methods.

**Conclusion:**

GATROF provides a new integrated predictive framework for predicting potential microbe–drug associations. By combining heterogeneous biological information, GAT-based topological representation learning, and Rotation Forest classification, GATROF may help prioritize candidate drug–microbe associations for further biological validation.

## Introduction

Studies have shown that the human body harbors a wide variety of microbial communities, including bacteria, fungi, viruses, and other microorganisms (Human Microbiome Project Consortium, 2012). These microbial communities are mainly distributed in the skin, oral cavity, gastrointestinal tract, reproductive system, and other regions of the human body. These niches are closely related to human health and are crucial for many physiological processes, including immune regulation, vitamin production, and maintenance of digestive function (Thiele et al., 2013). However, under certain conditions, some microorganisms may be associated with disease development of disease. For example, an imbalance in gut bacteria in the human gut microbiota may increase the risk of hypertension (Yu et al., 2026).

In recent years, the overuse and inappropriate use of antibiotics, mutations in microbial genes and horizontal gene transfer, and the spread of microbes in medical and social environments have led to antibiotic resistance in microbes. This has reduced the efficacy of otherwise effective antibiotic therapies ineffective and posed serious challenges to clinical treatment.

To address microbial resistance, effective computational models need to be developed to predict microbial drug resistance and identify new antibiotics. Such models can predict microbe–drug associations, thereby providing a simple and effective approach to addressing microbial resistance (Chen et al., 2025). Therefore, as an important strategy for addressin microbial resistance, the demand for efficient computational models has been increasing.

To date, researchers have used several microbe–drug association databases, including MDAD (Andersen et al., 2020), aBiofilm (Sun et al., 2018), and DrugVirus (Rajput et al., 2018), to construct a variety of predictive models for identifying potential microbe–drug associations. For example, in 2019, to predict potential links between microbes and drugs, Zhu et al. (Zhu et al., 2019) developed the HMDAKATZ prediction model based on the KATZ measure. In 2021, Deng et al. (Deng et al., 2022) designed a method named Graph2MDA, which inferred potential microbe–drug associations by constructing a multi-view attribute graph as the input to a variational graph autoencoder, thereby capturing both node-level and whole-graph information. Long et al. (Long and Luo, 2021) learned low-dimensional embedding representations of microbes and drugs using the metapath2vec scheme, designed a partially bipartite network projection recommendation algorithm, and proposed a computational method called HNERMDA. In 2023, Ma (Ma et al., 2023) combined a Graph Attention Network with a CNN-based classifier to construct a model termed GACNNMDA. Huang et al. (Huang et al., 2023) designed a model named GNAEMDA, which predicts microbe–drug associations based on a graph-normalized convolutional network. Cheng et al. (Cheng et al., 2022) designed a model termed NIRBMMDA, which predicts microbe–drug associations based on Neighborhood Inference (NI) and Restricted Boltzmann Machines (RBM). Li et al. innovatively combined matrix factorization with a three-layer heterogeneous network to create a model named MFTLHNMDA for inferring microbe–drug associations. Qu et al. (2023) proposed an integrated framework for identifying potential virus–drug associations. Kuan et al. (2024) developed a microbe–drug association prediction model based on graph attention networks and a bilayer random forest.

More recently, HeTAN and MCL-DMD have further extended heterogeneous biomedical association prediction. HeTAN models higher-order triplet relationships in heterogeneous biomedical networks through triplet-level attention (Tanvir et al., 2024), whereas MCL-DMD applies multi-modal contrastive learning to drug–microbe–disease association prediction by integrating heterogeneous graph information with domain-specific biomedical knowledge (Say et al., 2025). These studies suggest that heterogeneous graph modeling and multi-modal representation learning are important directions for biomedical association prediction.

To better exploit complementary information in sparse microbe–drug association data, we developed GATROF as a new integrated predictive framework by combining GAT with Rotation Forest. In GATROF, we first comprehensively considered multiple features of microbes and drugs, and then used a combination strategy to obtain integrated features of microbes and drugs respectively. Next, these integrated features were used by the GAT to learn complementary topology-aware representations of microbes and drugs. GATROF is not designed to rely solely on graph representation learning; instead, GAT-derived embeddings are integrated with association profiles, biological similarity features, disease-derived similarities, and RWR-based diffusion features. Finally, considering that ensemble classifiers generally produce more stable predictive results than single classifiers, the Rotation Forest was adopted to process the fused feature representation for final prediction, and the results generated by the Rotation Forest describe the likelihood score of interaction for each drug–microbe pair, in which, the drug–microbe pairs with high predicted scores would be regarded as the most likely to be associated among all predicted samples. Furthermore, we conducted extensive case studies and comparative experiments to evaluate the predictive performance of GATROF. Accordingly, GATROF achieved satisfactory results in predicting potential microbe–drug relationships and outperformed existing representative competing methods.

## Data sources

First, the relevant microbe–drug associations were obtained from the MDAD database (http://www.chengroup.cumt.edu.cn/MDAD/). This database contains 2,470 validated microbe–drug associations involving 1,373 drugs and 173 microbes. After rechecking the dataset, the MDAD benchmark dataset used in this study was consistently defined as containing 1,373 drugs, 173 microbes, and 2,470 known drug–microbe associations, and the drug–microbe association matrix was defined as A ∈ R^{1373 × 173}, where Aij = 1 denotes a known association between drug di and microbe mj, and Aij = 0 denotes an unknown or unlabeled pair. Subsequently, additional drug–disease and microbe–disease association data were obtained from the dataset of Wang et al. This dataset contains 70,315 reported drug–disease links and 15,633 reported microbe–disease links. Only disease-related associations explicitly linked to drugs or microbes present in MDAD were retained. Through this screening process, we retained 1,121 drug–disease associations involving 233 drugs and 109 diseases, as well as 402 microbe–disease associations involving 73 microbes and 109 diseases. Disease-related information was used only as auxiliary similarity information for feature construction rather than as the direct prediction target. Finally, we collected 138 known microbe–microbe interactions involving 123 bacteria from MDAD, as well as 5,586 known drug–drug associations involving 1,228 drugs from the dataset compiled by Deng et al. (Li et al., 2023). Details of these datasets are summarized in Table 1 below.

**TABLE 1:** Details of the downloaded datasets.

Type	Links	Microbes	Drugs	Diseases
Microbe–disease associations	402	73	-	109
Microbe–drug associations	2470	173	1373	-
Drug–disease associations	1121	-	233	109
Drug–drug associations	5586	-	1228	-
Microbe–microbe associations	138	123	-	-

## Methods

**FIGURE 1:**
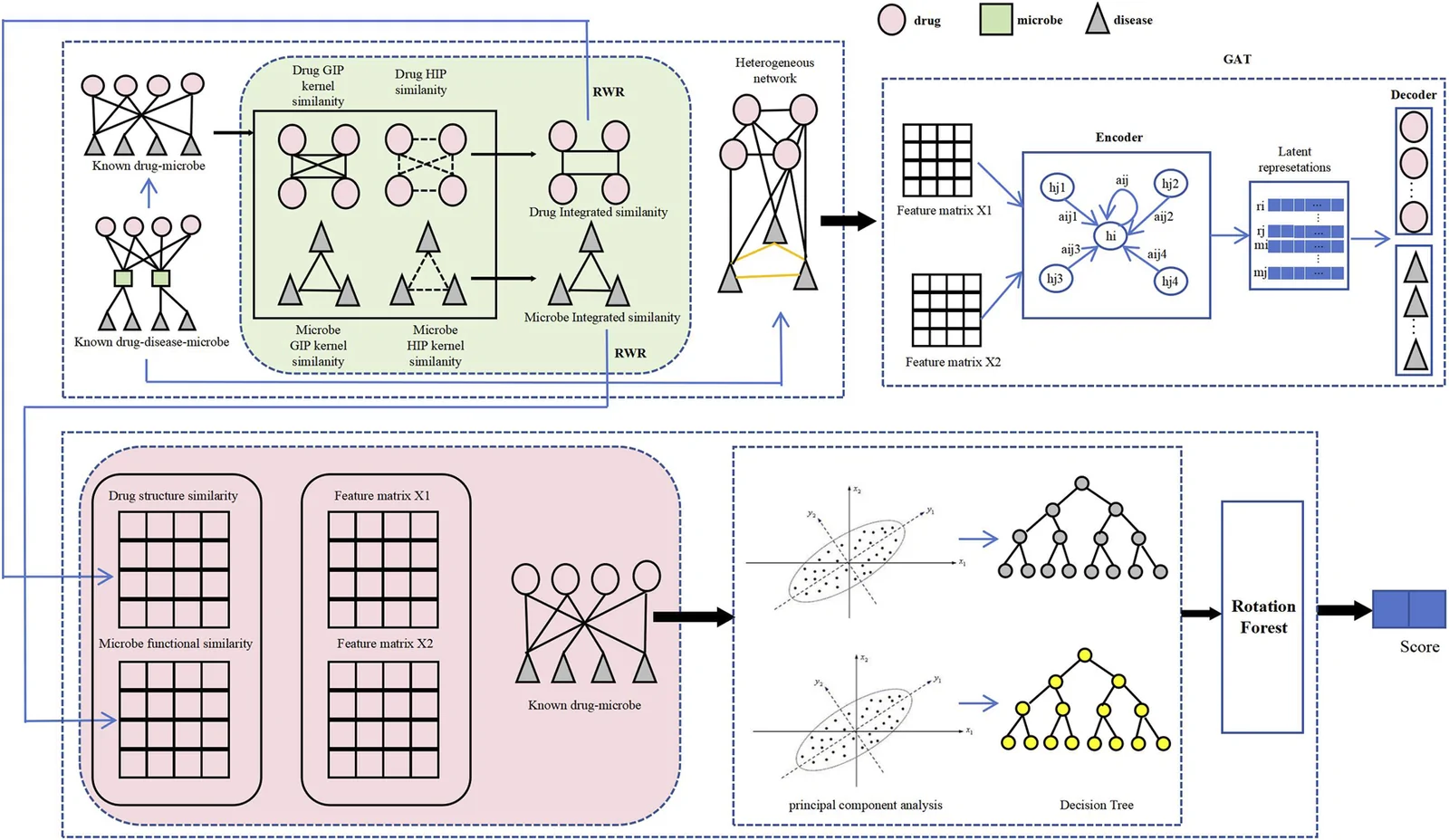
Flowchart of GATROF.

As shown in Figure 1, GATROF consists of the following three major components:

Part 1: Using newly downloaded data of microbes, drugs, and diseases to construct the microbe–drug association network HN1.

Part 2: Based on diverse biological feature data for microbes and drugs, two feature matrices were created for microbes and drugs separately, and then, these two feature matrices together with the microbe–drug network would be fed into the GAT to learn low-dimensional feature representations for microbes and drugs respectively.

Part 3: These two newly obtained low-dimensional feature representations together with these two feature matrices were input into the Rotation Forest model for final prediction, by which, the probability scores of drug–microbe associations would be calculated.

### Construction of the heterogeneous microbe–drug network

In this section, the heterogeneous drug–microbe network is constructed from the retained drugs, microbes, and known drug–microbe associations. Let $R=\left\{r_{1},r_{2},\ldots ,r_{n_{r}}\right\}$ denote the set of drugs and let $M=\left\{m_{1},m_{2},\ldots ,m_{n_{m}}\right\}$ denote the set of microbes, where $n_{r}$ and $n_{m}$ are the numbers of drugs and microbes, respectively. The known drug–microbe association matrix is denoted as $B\in {\left\{0,1\right\}}^{n_{r}\times n_{m}}$ where each row corresponds to a drug and each column corresponds to a microbe:

$$B_{\text{ij}}=\left\{\begin{array}{l} 1,\text{if drug}r_{i}\text{is known to be associated with microbe}m_{j},\\ 0,\text{otherwise}. \end{array}\right.$$ (1)

In Equation 1, $B\left(i,:\right)$ denotes the interaction profile of drug $r_{i}$ , and $B\left(:,j\right)$ denotes the interaction profile of microbe $m_{j}$ .

For any two drugs $r_{i}$ and $r_{j}$ , the Gaussian interaction profile (GIP) kernel similarity is calculated as follows:

$$S_{r}^{\text{GIP}}\left(i,j\right)=\exp\left(‐\gamma_{r}{\left\|B\left(i,:\right)‐B\left(j,:\right)\right\|}_{2}^{2}\right)$$ (2)

$$\gamma_{r}=\frac{n_{r}}{\sum_{i=1}^{n_{r}}{\left\|B\left(i,:\right)\right\|}_{2}^{2}}$$ (3)

Similarly, for any two microbes $m_{i}$ and $m_{j},$ the GIP kernel similarity is calculated as follows:

$$S_{m}^{\text{GIP}}\left(i,j\right)=\exp\left(‐\gamma_{m}{\left\|B\left(:,i\right)‐B\left(:,j\right)\right\|}_{2}^{2}\right)$$ (4)

$$\gamma_{m}=\frac{n_{m}}{\sum_{j=1}^{n_{m}}{\left\|B\left(:,j\right)\right\|}_{2}^{2}}$$ (5)

Based on Equations 2–5, the drug GIP similarity matrix $S_{r}^{GIP}\in R^{n_{r}\times n_{r}}$ and the microbe GIP similarity matrix $S_{m}^{GIP}\in R^{n_{m}\times n_{m}}$ can be obtained.

To further measure interaction-profile differences, the Hamming interaction profile (HIP) similarity is introduced. For any two drugs $r_{i}$ and $r_{j}$ , the HIP similarity is defined as follows:

$$S_{r}^{\text{HIP}}\left(i,j\right)=1‐\frac{1}{n_{m}}\sum_{k=1}^{n_{m}}1\left(B_{\text{ik}}\neq B_{\text{jk}}\right).$$ (6)

Similarly, for any two microbes $m_{i}$ and $m_{j}$ , the HIP similarity is calculated as follows:

$$S_{m}^{\text{HIP}}\left(i,j\right)=1‐\frac{1}{n_{r}}\sum_{k=1}^{n_{r}}1\left(B_{\text{ki}}\neq B_{\text{kj}}\right).$$ (7)

In Equations 6, 7, $1\left(\cdot \right)$ is the indicator function. The denominators $n_{m}$ and $n_{r}$ correspond to the lengths of drug and microbe interaction profiles, respectively.

Let $G_{r}\in {\left\{0,1\right\}}^{n_{r}\times n_{r}}$ and $G_{m}\in {\left\{0,1\right\}}^{n_{m}\times n_{m}}$ denote the known drug–drug and microbe–microbe interaction matrices, respectively. Therefore, based on Equations 2–7, the integrated drug similarity matrix $S_{r}$ is defined as follows:

$$S_{r}\left(i,j\right)=\left\{\begin{array}{l} 1,\text{if }i=j\text{ or }G_{r}\left(i,j\right)=1,\\ \frac{S_{r}^{\text{GIP}}\left(i,j\right)+S_{r}^{\text{HIP}}\left(i,j\right)}{2},\text{otherwise}. \end{array}\right.$$ (8)

Similarly, the integrated microbe similarity matrix $S_{m}$ is defined as follows:

$$S_{m}\left(i,j\right)=\left\{\begin{array}{l} 1,\text{ifi}=\text{jor}G_{m}\left(i,j\right)=1\\ \frac{S_{m}^{\text{GIP}}\left(i,j\right)+S_{m}^{\text{HIP}}\left(i,j\right)}{2},\text{otherwise} \end{array}\right.$$ (9)

Therefore, based on Equations 8, 9, the heterogeneous drug–microbe network matrix H is constructed as follows:

$$H=\left[\begin{array}{cc} S_{r} & B\\ B^{T} & S_{m} \end{array}\right]\in R^{\left(n_{r}+n_{m}\right)\times \left(n_{r}+n_{m}\right)}$$ (10)

Based on Equation 10, a heterogeneous graph $G=\left(V,E\right)$ can be constructed, where $V=V_{r}\cup V_{m}$ , $\left|V_{r}\left|=\right.n_{r}\right.$ , $\left|V_{m}\left|=\right.n_{m}\right.$ , and $\left|V\left|=\right.n_{r}+n_{m}\right.$ . An edge exists between nodes $v_{i}$ and $v_{j}$ if and only if $H_{ij}$ is nonzero.

## Low-dimensional feature extraction of microbes and drugs based on GAT

### Architecture of the GAT

The Graph Attention Network (GAT) is used to learn low-dimensional topological representations of drug and microbe nodes from the heterogeneous graph G constructed from Equation 10. The initial node feature matrix is set as $X^{\left(0\right)}=H$ , where the i -th row of H is used as the initial feature representation of node $v_{i}$ . Let $h_{i}^{\left(l\right)}$ denote the representation of node $v_{i}$ in the l -th GAT layer, and let $N_{i}$ denote the neighbor set of $v_{i}$ in G .

Step 1 (Encoder): For any given node $v_{i}$ in G and any neighbor $v_{j}\in N_{i}$ , GAT first computes the unnormalized attention score between $v_{i}$ and $v_{j}$ as follows:

$$e_{ij}^{\left(l\right)}=LeakyReLU\left({\left(a^{\left(l\right)}\right)}^{T}\left[W^{\left(l\right)}h_{i}^{\left(l\right)}\|W^{\left(l\right)}h_{j}^{\left(l\right)}\right]\right).$$ (11)

In Equation 11, $W^{\left(l\right)}$ is a trainable weight matrix, $a^{\left(l\right)}$ is a trainable attention vector, and ‖ denotes vector concatenation. Based on Equation 11, the attention coefficients are normalized over the neighbor set of node $v_{i}$ by the softmax function:

$$\alpha_{ij}^{\left(l\right)}=\frac{\exp\left(e_{ij}^{\left(l\right)}\right)}{\sum_{k\in N_{i}}\exp\left(e_{ik}^{\left(l\right)}\right)}$$ (12)

Based on Equation 12, the new representation of node $v_{i}$ is obtained by aggregating the transformed features of its neighboring nodes with the learned attention weights:

$$h_{i}^{\left(l+1\right)}=\sigma \left(\sum_{j\in N_{i}}\alpha_{ij}^{\left(l\right)}W^{\left(l\right)}h_{j}^{\left(l\right)}\right)$$ (13)

where $\sigma \left(\cdot \right)$ is a nonlinear activation function. Therefore, based on Equations 11–13, the encoder produces the final node embedding matrix:

$$Z=\left[\begin{array}{c} Z_{r}\\ Z_{m} \end{array}\right]\in R^{\left(n_{r}+n_{m}\right)\times d_{z}}$$ (14)

In Equation 14, $Z_{r}\in R^{n_{r}\times d_{z}}$ and $Z_{m}\in R^{n_{m}\times d_{z}}$ denote the low-dimensional topological representations of drug nodes and microbe nodes, respectively.

Step 2 (Decoder): Based on the embedding matrix Z obtained from Equation 14, an inner-product decoder is used to reconstruct the heterogeneous network matrix:

$$\hat{H}=sigmoid\left(ZZ^{T}\right)$$ (15)

$$sigmoid\left(x\right)=\frac{1}{1+\exp\left(‐x\right)}$$ (16)

Here, $\hat{H}$ denotes the reconstructed heterogeneous network matrix. Based on Equations 15, 16, the decoder estimates the connection strength between any two nodes according to the inner product of their learned embeddings.

Step 3 (Optimization): Considering that the reconstructed matrix $\hat{H}$ should be close to the original heterogeneous network matrix H , the GAT module is optimized by minimizing the following reconstruction loss:

$$L_{GAT}=\frac{{\left\|\hat{H}‐H\right\|}_{F}^{2}}{{\left(n_{r}+n_{m}\right)}^{2}}$$ (17)

In Equation 17, $\|\;{\|}_{F}$ denotes the Frobenius norm. The loss in Equation 17 is used only to train the GAT-based topological representation module, whereas the final drug–microbe association prediction is performed by the Rotation Forest classifier using the constructed pairwise features.

## Construction of the microbe and drug feature matrices

Multiple biological feature sources are constructed and integrated to form the drug-side and microbe-side feature matrices. The drug structural similarity matrix $S_{r}^{che}$ is obtained from SIMCOMP2, which measures drug similarity based on chemical structure information (Zhu et al., 2021). The drug structural similarity information downloaded from SIMCOMP2 is also used in the subsequent construction of the drug feature matrix. The microbial functional similarity matrix $S_{m}^{fun}$ is obtained using the method proposed by Kamneva (Long et al., 2020). In the microbial protein–protein functional association network, nodes represent gene families encoded by microbial genomes and edges denote genetic-neighbor association scores based on the STRING database. The functional similarity between two microbes is calculated from the link scores connecting their microbial gene families relative to the total link scores of the two microbial gene-family sets.

To obtain topology-aware diffusion features, random walk with restart (RWR) is performed on the integrated drug similarity matrix $S_{r}$ and the integrated microbe similarity matrix $S_{m}$ . The transition matrices are obtained by row normalization:

$$Q_{r}\left(i,j\right)=\frac{S_{r}\left(i,j\right)}{\sum_{j=1}^{n_{r}}S_{r}\left(i,j\right)}$$ (18)

$$Q_{m}\left(i,j\right)=\frac{S_{m}\left(i,j\right)}{\sum_{j=1}^{n_{m}}S_{m}\left(i,j\right)}$$ (19)

Based on Equations 18, 19, the RWR update rules for drug $r_{i}$ and microbe $m_{i}$ are defined in Equations 20, 21, respectively:

$$q_{r_{i}}^{\left(t+1\right)}=\lambda q_{r_{i}}^{\left(t\right)}Q_{r}+\left(1‐\lambda \right)e_{r_{i}}$$ (20)

$$q_{m_{i}}^{\left(t+1\right)}=\lambda q_{m_{i}}^{\left(t\right)}Q_{m}+\left(1‐\lambda \right)e_{m_{i}}$$ (21)

In the above equations, $Q_{r}$ and $Q_{m}$ are transition probability matrices. $e_{r_{i}}$ and $e_{m_{i}}$ are the initial probability vectors of drug $r_{i}$ and microbe $m_{i}$ , respectively, and $q_{r_{i}}^{\left(t\right)}$ and $q_{m_{i}}^{\left(t\right)}$ denote the probability vectors at the t -th iteration. Based on the above RWR process, the RWR-based topological matrices $R_{r}^{RWR}\in R^{n_{r}\times n_{r}}$ and $R_{m}^{RWR}\in R^{n_{m}\times n_{m}}$ can be obtained.

In addition, let $n_{s}$ denote the number of newly retained diseases. Similar to the construction of the drug–microbe adjacency matrix, the drug–disease association matrix $D_{r}\in {\left\{0,1\right\}}^{n_{r}\times n_{s}}$ and the microbe–disease association matrix $D_{m}\in {\left\{0,1\right\}}^{n_{m}\times n_{s}}$ can be obtained from the downloaded disease-related associations. Then, for any two given drug nodes $r_{i}$ and $r_{j}$ , their disease-based cosine similarity is calculated as follows:

$$S_{r}^{dis}\left(i,j\right)=\frac{D_{r}\left(i,:\right)\cdot D_{r}\left(j,:\right)}{{\left\|D_{r}\left(i,:\right)\right\|}_{2}{\left\|D_{r}\left(j,:\right)\right\|}_{2}+\varepsilon }$$ (22)

where $D_{r}\left(i,:\right)$ denotes the i -th row of $D_{r}$ . Similarly, for any two given microbe nodes $m_{i}$ and $m_{j}$ , their disease-based cosine similarity is calculated as follows:

$$S_{m}^{dis}\left(i,j\right)=\frac{D_{m}\left(i,:\right)\cdot D_{m}\left(j,:\right)}{{\left\|D_{m}\left(i,:\right)\right\|}_{2}{\left\|D_{m}\left(j,:\right)\right\|}_{2}+\varepsilon }$$ (23)

In Equations 22, 23, ε is a small positive constant used to avoid division by zero.

Let $A_{r}^{attr}\in R^{n_{r}\times d_{r}}$ and $A_{m}^{attr}\in R^{n_{m}\times d_{m}}$ denote the drug and microbe attribute representation matrices constructed from the available biological attributes. Based on the drug topological representation matrix $Z_{r}$ , drug attribute representation matrix $A_{r}^{attr}$ , drug structural similarity matrix $S_{r}^{che}$ , drug disease-based cosine similarity matrix $S_{r}^{dis}$ , RWR-based drug similarity matrix $R_{r}^{RWR}$ , and the drug–microbe adjacency matrix B , and inspired by Xuan et al. (Hattori et al., 2010), the new drug feature matrix B_r is constructed as follows:

$$B_{r}=\left[Z_{r}\;\|\;A_{r}^{attr}\;\left\|\;S_{r}^{che}\;\right\|\;S_{r}^{dis}\;\left\|\;R_{r}^{RWR}\;\right\|\;B\right]$$ (24)

Similarly, based on the microbial topological representation matrix $Z_{m}$ , microbe attribute representation matrix $A_{m}^{attr}$ , microbe functional similarity matrix $S_{m}^{fun}$ , microbe disease-based cosine similarity matrix $S_{m}^{dis}$ , RWR-based microbe similarity matrix $R_{m}^{RWR}$ , and the transposed drug–microbe adjacency matrix $B^{T}$ , the new microbe feature matrix $B_{m}$ is constructed as follows:

$$B_{m}=\left[Z_{m}\;\|\;A_{m}^{attr}\;\left\|\;S_{m}^{fun}\;\right\|\;S_{m}^{dis}\;\left\|\;R_{m}^{RWR}\;\right\|\;B^{T}\right]$$ (25)

In Equations 24, 25, ‖ denotes horizontal concatenation. All matrices in Equation 24 have $n_{r}$ rows, and all matrices in Equation 25 have $n_{m}$ rows; therefore, the two feature matrices are dimensionally well defined. Specifically,

$$B_{r}\in R^{n_{r}\times p_{r}},p_{r}=d_{z}+d_{r}+3n_{r}+n_{m}$$ (26)

$$B_{m}\in R^{n_{m}\times p_{m}},p_{m}=d_{z}+d_{m}+3n_{m}+n_{r}$$ (27)

For any given drug $r_{i}$ and microbe $m_{j}$ , based on Equations 24–27, the pairwise drug–microbe feature vector is obtained by concatenating the corresponding drug-side and microbe-side representations, and its dimension is given in Equation 29:

$$x_{ij}=B_{r}(i\left.,:)\right\|B_{m}\left(j,:\right)$$ (28)

$$x_{ij}\in R^{p_{r}+p_{m}}$$ (29)

The obtained pairwise feature vectors are then used as the input of PCA and Rotation Forest. Since the numbers of retained drugs, microbes, and biological feature sources may vary across datasets and implementations, the feature dimension before PCA is denoted generally as p .

In this feature construction strategy, the GAT-derived topological representations are only one component of the final pairwise representation. They are concatenated with engineered biological similarity features, association-profile features, disease-derived similarities, and RWR-based diffusion features; therefore, the final classifier input reflects heterogeneous feature fusion rather than a purely GAT-based embedding.

## Architecture of rotation forest

Traditional machine learning may encounter shortcomings such as overfitting and the inability to provide uncertainty estimates for predictions when facing complex nonlinear patterns. To calculate the potential scores of latent drug–microbe relationships, we construct a Rotation Forest model in this section and treat the drug–microbe problem as a binary classification task. By selecting features within Rotation Forest, the model performance can be improved and the risk of overfitting can be reduced. The procedure is described as follows.

Step 1 (Input construction and dimensionality reduction): Let Ω denote the set of selected positive and pseudo-negative drug–microbe pairs.
Step 1.1: Based on Equation 28, the pairwise feature vectors are collected to form the classifier input matrix, and the corresponding labels are defined in Equation 30:

Where $N=\left|\Omega \right|$ is the number of samples and $p=p_{r}+p_{m}$ is the feature dimension before dimensionality reduction.

$$X={\left[x_{ij}\right]}_{\left(i,j\right)\in \Omega }\in R^{N\times p},y\in {\left\{0,1\right\}}^{N}$$ (30)

Step 1.2: Before classification, PCA is used to reduce redundant information in X , as shown in Equation 31:

$$\tilde{X}=XW_{q}$$ (31)

where $W_{q}\in R^{p\times q}$ is the PCA projection matrix and $\tilde{X}\in R^{N\times q}$ . In this study, the PCA-reduced dimension was set to q=954 according to the original experimental setting.

Step 2 (Construction of rotation matrices): Rotation Forest is then used as the final classifier. Let $D_{1},D_{2},\ldots ,D_{T}$ denote T base decision-tree classifiers.
Step 2.1: For the t -th base classifier, the q -dimensional feature set is randomly divided into K non-overlapping subsets:

$$F_{t,1},F_{t,2},\ldots ,F_{t,K}$$ (32)

Step 2.2: For each feature subset $F_{t,k}$ , the corresponding feature submatrix is extracted from $\tilde{X}$ . Following the original Rotation Forest procedure, a bootstrap sample containing a fixed proportion of training samples can be drawn from this submatrix to increase classifier diversity.
Step 2.3: PCA is performed on each subset-specific sample to obtain a local rotation matrix $C_{t,k}$ . Therefore, based on Equation 32, the local rotation matrices are assembled into a block-diagonal rotation matrix:

$$R_{t}=diag\left(C_{t,1},C_{t,2},\ldots ,C_{t,K}\right)$$ (33)

Step 2.4: Based on Equation 33, the transformed training matrix for the t -th base classifier is obtained as follows:

$${\tilde{X}}^{\left(t\right)}=\tilde{X}R_{t}$$ (34)

The transformed matrix ${\tilde{X}}^{\left(t\right)}$ is then used to train the base classifier $D_{t}$ .

Step 3 (Prediction and score calculation): For a candidate drug–microbe pair represented by ${\tilde{x}}_{ij}$ , the trained Rotation Forest model calculates its association probability as follows.
Step 3.1: The candidate feature vector is first transformed by the rotation matrix of each base classifier:

$${\tilde{x}}_{ij}^{\left(t\right)}={\tilde{x}}_{ij}R_{t}$$ (35)

Step 3.2: Based on Equations 34, 35, each base classifier outputs the probability that the candidate pair belongs to class c , and the final class probability is obtained by averaging the outputs of all base classifiers:

$$P\left(c|{\tilde{x}}_{ij}\right)=\frac{1}{T}\sum_{t=1}^{T}P_{t}\left(c|{\tilde{x}}_{ij}^{\left(t\right)}\right),c\in \left\{0,1\right\}$$ (36)

Step 3.3: Finally, based on Equation 36, the association score between drug $r_{i}$ and microbe $m_{j}$ is defined in Equation 37 as the predicted probability of the positive class:

$$Score_{ij}=P\left(y_{ij}=1|{\tilde{x}}_{ij}\right)$$ (37)

A larger $Score_{ij}$ indicates a higher probability that drug $r_{i}$ is associated with microbe $m_{j}$ .

## Parameter settings and computational complexity

The parameters were optimized by grid search. In this study, the number of feature subsets K and the number of base classifiers T in Rotation Forest were set to 100 and 200, respectively. Here, T corresponds to n_classifier in the implementation.

The Rotation Forest classifier consists of multiple weak decision-tree classifiers. For each base classifier, the feature set is randomly divided into several low-dimensional subsets, and PCA is applied to each subset to generate the corresponding rotation matrix. Since each base classifier is trained on a rotated feature representation, the training process is similar to that of a decision tree. Its approximate time complexity is expressed in Equation 38:

$$O\left(n|D\left|\log\right|\left.D\right|\right)$$ (38)

where n denotes the number of training samples and $\left|D\right|$ denotes the feature dimensionality.

In the complete GATROF pipeline, the main computational costs are introduced by similarity calculation, RWR-based feature construction, GAT-based representation learning, repeated PCA transformations, and Rotation Forest classification. For larger datasets, dense similarity matrices and repeated PCA operations may become the main scalability bottlenecks. These costs can be further reduced by sparse matrix representation, approximate similarity calculation, mini-batch graph learning, and parallel ensemble training.

## Results

To optimize the model, we first examined the effects of relevant factors on the predictive performance of GATROF. We then compared GATROF with seven state-of-the-art competing prediction techniques. Finally, to illustrate the efficiency of GATROF, several specific drugs and microbes were used as case studies.

## Hyperparameter analysis

From the above descriptions, GATROF contains several important hyperparameters, including the learning rate and dropout rate of the GAT module, the feature subset parameter K in Rotation Forest, and the number of base classifiers n_classifier. To evaluate their influence, we performed five-fold stratified cross-validation on MDAD and used AUC as the primary selection criterion. AUPR and F1-score were also considered because microbe-drug association prediction is affected by class imbalance. In each fold, positive associations in the test fold were removed from the interaction matrix before feature construction, and feature normalization and PCA were fitted only on the training fold.

**FIGURE 2:**
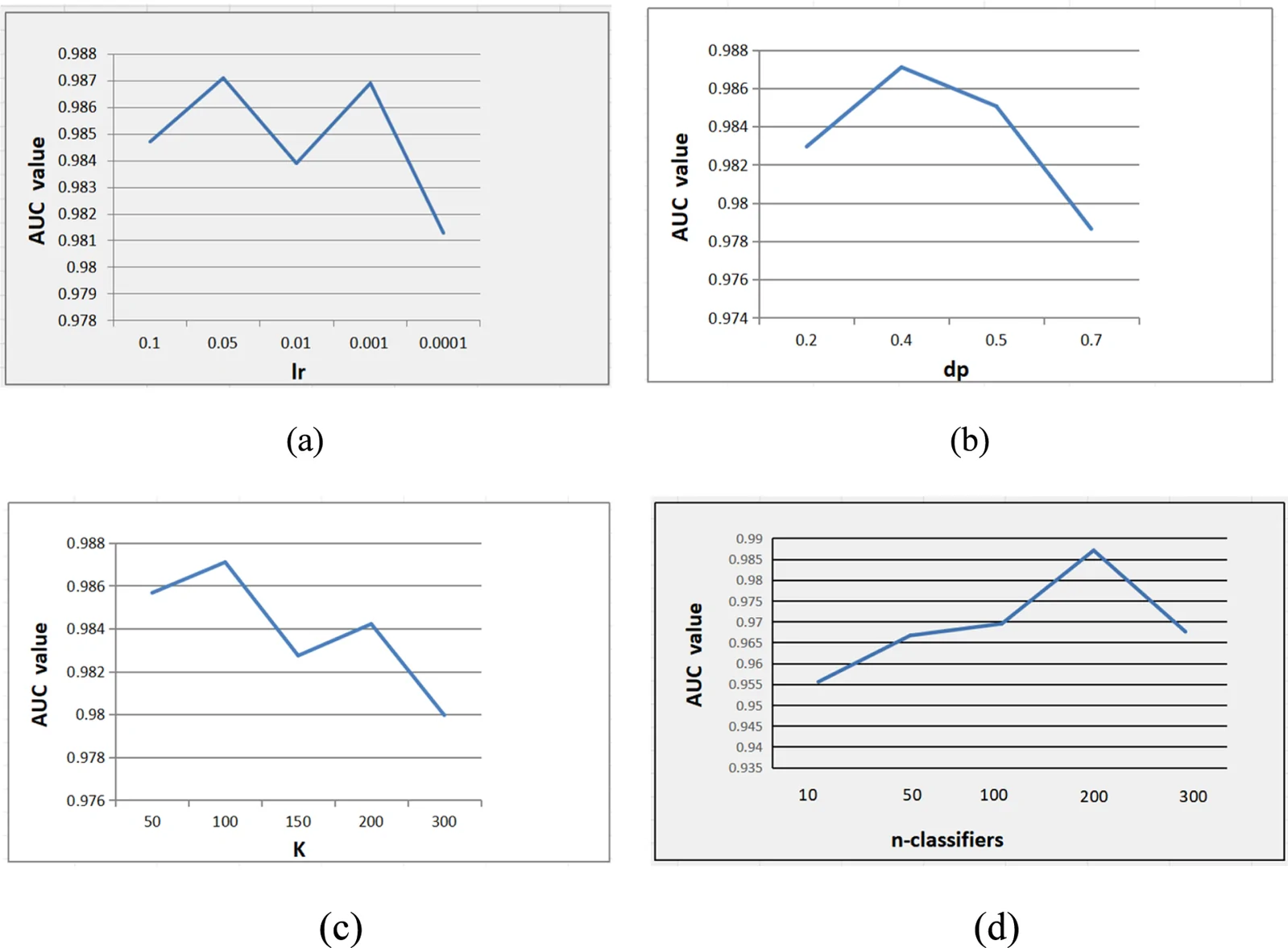
Influence of hyperparameters on the predictive performance of GATROF. **(a,b)** Show the AUC values of GATROF under different learning rates and dropout rates in GAT, respectively. **(c,d)** Illustrate the AUC values of GATROF under different K values in the Rotation Forest model and the contribution values of different numbers of member classifiers n_classifier, respectively.

For simplicity, lr, dp, K, and n_classifier denote the learning rate, dropout rate, feature subset parameter, and number of base classifiers, respectively. The learning rate was searched in {0.0001, 0.001, 0.01, 0.05, 0.1}. As can be clearly seen from Figure 2a, when lr was set to 0.01, GATROF achieved the highest AUC value. Next, dp was restricted to the range {0.2, 0.4, 0.5, 0.7}. As shown in Figure 2b, GATROF achieved the highest AUC value when dp was set to 0.4. In addition, K was restricted to the range {50, 100, 200, 300}. As shown in Figure 2c, GATROF achieved the highest AUC value when K was set to 100. Finally, n_classifier was restricted to the range {10, 50,100, 200, 300}. When l was set to 0.0012, the performance of GATROF reached its optimum, as shown in Figure 2d.

In addition to the main GATROF parameters, we also briefly examined the PCA dimension and tree-related hyperparameters in the controlled supplementary experiments using the Random Forest variant. The PCA dimension controls the compressed feature size before classification, whereas the number of trees, maximum tree depth, and minimum samples per leaf affect model stability and complexity. The supplementary results showed that pca_dim = 128 and n_estimators = 300 provided a stable balance between predictive performance and computational cost, while max_depth = None and min_samples_leaf = 1 were retained as effective default settings.

## Comparison with the latest methods

To further verify the predictive performance of GATROF, we compared it with seven competing methods based on MDAD samples.

◆LAGCN is a computational model that infers unknown drug–disease associations based on a graph convolutional network and an attention mechanism.

◆GSAMDA is a model based on a Graph Attention Network and a sparse autoencoder.

◆HMDAKATZ (Xuan et al., 2020) is a computational method based on the KATZ model for predicting potential microbe–drug associations.

◆KATZHMDA (Rodriguez et al., 2006) is a path-based prediction model for microbe–disease associations.

◆SCSMDA (Wang et al., 2022) predicts microbe–drug associations through structure-enhanced contrastive learning and a self-paced negative-sampling strategy.

◆MHBVDA is an ensemble computational framework for inferring microbe-drug associations by integrating heterogeneous graph inference, bounded nuclear norm regularization, and ensemble learning on integrated drug and microbe similarity networks.

◆NIRBMMDA predicts potential microbe-drug associations by combining neighborhood-based inference and restricted Boltzmann machine modules through an ensemble strategy, using known associations together with integrated microbe and drug similarity information.

**FIGURE 3:**
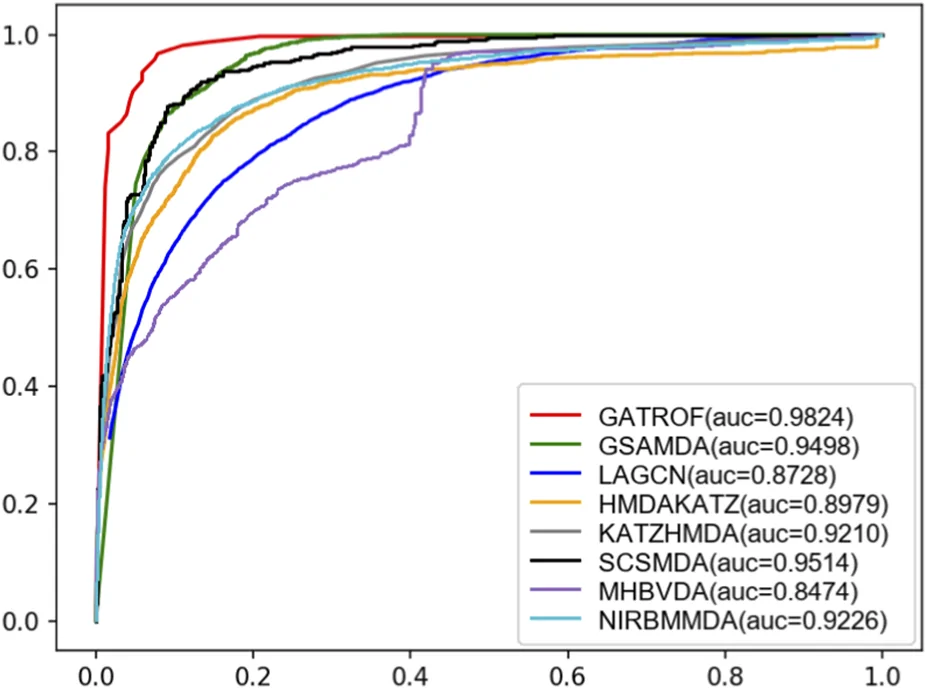
ROC curves obtained by competing techniques based on MDAD.

To verify the predictive performance of GATROF, we compared it with seven competing methods based on MDAD samples in this section. In our tests, AUC, accuracy, and F1-score were used as performance metrics. The quantitative results are summarized in Table 2, and the corresponding ROC curves are shown in Figure 3. As can be clearly seen from Table 2, GATROF achieved the highest AUC value of 0.9824 ± 0.0012, whereas SCSMDA ranked second with an AUC value of 0.9514 ± 0.0031, and MHBVDA obtained the lowest AUC value of 0.8474. In terms of accuracy and F1-score, GATROF achieved the highest values of 0.9955 and 0.7106, respectively. Therefore, our model outperformed these five competing models.

**TABLE 2:** Comparison of the AUC, accuracy, and F1-score values obtained by GATROF and seven competing methods under 10-fold CV based on MDAD.

Method	AUC (10-fold)	Accuracy	F1-score
LAGCN	0.8728 ± 0.0042	0.9413	0.1838
GSAMDA	0.9498 ± 0.0003	0.9896	0.6433
HMDAKATZ	0.8979 ± 0.0023	0.9876	0.6959
KATZHMDA	0.9210 ± 0.0037	0.9884	0.7016
SCSMDA	0.9514 ± 0.0031	0.9365	0.2594
MHBVDA	0.8474	0.7620	0.8000
NIRBMMDA	0.9226	0.8542	0.8510
GATROF	0.9843 ± 0.0034	0.9908 ± 0.0016	0.9494 ± 0.0082

### Additional experimental analysis

To further examine the effectiveness and robustness of the proposed framework, we conducted three additional analyses, including feature-source ablation analysis, classifier sensitivity analysis, and positive-to-negative sample ratio analysis. All additional experiments were performed using five-fold stratified cross-validation, and the results are reported as mean ± standard deviation.

### Feature-source ablation analysis

To quantify the contribution of different feature sources, we performed a feature-source ablation study under the same five-fold stratified cross-validation protocol used in the additional experiments. In each fold, the training and test pairs were separated before feature construction. The positive associations in the test fold were masked from the drug–microbe association matrix, and feature scaling and PCA were fitted only on the training data to avoid information leakage. To ensure comparability among different ablation settings, the same downstream PCA and Rotation Forest configuration was used for all feature variants.

Table 3 summarizes the ablation results. The full feature setting achieved stable and balanced performance, with an AUC of 0.9843 ± 0.0034, an AUPR of 0.9908 ± 0.0016, an Accuracy of 0.9494 ± 0.0082, and an F1-score of 0.9603 ± 0.0067. Removing similarity features reduced the AUC to 0.9735 ± 0.0070 and also decreased Accuracy and F1-score, indicating that structural and functional similarity information provides important predictive signals. Removing association-profile features caused only a small decrease in AUC but led to clearer reductions in Accuracy and F1-score, suggesting that known drug–microbe association profiles remain important for accurate classification.

**TABLE 3:** Feature-source ablation analysis under five-fold stratified cross-validation.

Setting	AUC	AUPR	Accuracy	F1
Full	0.9843 ± 0.0034	0.9908 ± 0.0016	0.9494 ± 0.0082	0.9603 ± 0.0067
without_assoc	0.9814 ± 0.0031	0.9888 ± 0.0019	0.9297 ± 0.0089	0.9453 ± 0.0072
without_similarity	0.9735 ± 0.0070	0.9846 ± 0.0030	0.9285 ± 0.0147	0.9433 ± 0.0119
without_disease	0.9846 ± 0.0032	0.9911 ± 0.0015	0.9480 ± 0.0064	0.9592 ± 0.0054
topo_only	0.9184 ± 0.0120	0.9482 ± 0.0098	0.8697 ± 0.0123	0.8973 ± 0.0101
assoc_only	0.9613 ± 0.0058	0.9757 ± 0.0038	0.9035 ± 0.0070	0.9218 ± 0.0064
similarity_only	0.9748 ± 0.0031	0.9787 ± 0.0023	0.9450 ± 0.0050	0.9574 ± 0.0039
disease_only	0.8372 ± 0.0170	0.8930 ± 0.0146	0.7616 ± 0.0245	0.8018 ± 0.0098
rwr_only	0.8941 ± 0.0122	0.9427 ± 0.0060	0.8097 ± 0.0116	0.8534 ± 0.0095

The removal of disease-derived features produced AUC and AUPR values close to those of the full feature setting. Because these differences were very small relative to the reported standard deviations, they should not be interpreted as a consistent performance gain after removing disease information. Instead, the results suggest that disease-derived features provide auxiliary information in this dataset but are not the dominant source of predictive performance. This interpretation is further supported by the disease-only setting, which showed the weakest performance among the single-source settings.

The single-source results further demonstrate that no individual feature source can fully replace the fused representation. The similarity-only setting retained relatively strong performance, whereas the topology-only, RWR-only, and disease-only settings showed obvious performance degradation. These observations indicate that association profiles and structural/functional similarities are the primary contributors, while GAT-derived topological representations, RWR-based diffusion features, and disease-derived similarities provide complementary information.

The limited standalone contribution of the GAT-derived representation may be attributed to feature redundancy among topology-related feature sources. Association profiles, GIP/HIP similarities, RWR-based diffusion features, and GAT embeddings all encode information derived directly or indirectly from the known drug–microbe association network. Therefore, GAT should be interpreted as a complementary topology-aware representation module rather than the dominant source of predictive performance.

Overall, the ablation results support the effectiveness of integrating heterogeneous biological and topological feature sources in GATROF.

## Classifier sensitivity analysis

To examine whether the fused features contain transferable discriminative information, we compared several classifiers using the same full-feature input and the same five-fold stratified cross-validation protocol. The preprocessing procedure, feature scaling, PCA setting, and data splits were kept consistent, and only the classifier was changed. This experiment was designed as a sensitivity analysis of the classifier module rather than as a replacement of the main GATROF framework.

As shown in Table 4, the ensemble classifiers generally performed better than Logistic Regression, indicating that the fused drug–microbe features contain nonlinear discriminative patterns. Random Forest obtained the highest AUC and Accuracy, with values of 0.9952 ± 0.0011 and 0.9610 ± 0.0048, respectively. GBDT achieved the highest AUPR of 0.9928 ± 0.0017. Rotation Forest achieved the highest F1-score of 0.9603 ± 0.0067 and maintained competitive AUC and AUPR values.

**TABLE 4:** Classifier sensitivity analysis under the same full-feature input.

Classifier	AUC	AUPR	Accuracy	F1
Random forest	0.9952 ± 0.0011	0.9892 ± 0.0006	0.9610 ± 0.0048	0.9597 ± 0.0037
GBDT	0.9885 ± 0.0022	0.9928 ± 0.0017	0.9480 ± 0.0022	0.9595 ± 0.0018
Rotation forest	0.9843 ± 0.0034	0.9908 ± 0.0016	0.9494 ± 0.0082	0.9603 ± 0.0067
Logistic regression	0.9129 ± 0.0123	0.9158 ± 0.0146	0.8880 ± 0.0104	0.9119 ± 0.0081

These results show that the proposed fused representation is effective across different nonlinear ensemble classifiers. Although Random Forest achieved a higher AUC in this sensitivity analysis, Rotation Forest still provided balanced classification performance and remained consistent with the original design of GATROF. Therefore, Rotation Forest was retained as the final prediction module in the proposed framework.

The classifier sensitivity results also suggest that further optimization of the classifier module may be a useful direction for future improvement, but they do not change the main conclusion that heterogeneous feature fusion and graph-based representation learning are effective for microbe–drug association prediction.

## Effect of positive-to-negative sample ratio

Since MDAD contains confirmed positive associations but no experimentally verified negative associations, pseudo-negative samples are usually selected from unlabeled drug-microbe pairs. To evaluate the influence of different sampling settings, we tested GATROF under different positive-to-negative sample ratios.

As shown in Table 5, the AUC remained stable across different ratios, ranging from 0.9893 to 0.9934, indicating that the model maintained strong ranking ability under different pseudo-negative sampling settings. However, AUPR and F1-score decreased when the number of negative samples increased, suggesting that class imbalance mainly affects precision-recall-related metrics and positive-class recall. Therefore, the positive-to-negative ratio should be carefully reported in microbe-drug association prediction tasks.

**TABLE 5:** Performance of GATROF under different positive-to-negative sample ratios.

Ratio	AUC	AUPR	Accuracy	F1
2:1	0.9893 ± 0.0032	0.9945 ± 0.0017	0.9452 ± 0.0048	0.9597 ± 0.0036
1:1	0.9912 ± 0.0012	0.9918 ± 0.0011	0.9518 ± 0.0035	0.9519 ± 0.0037
1:2	0.9929 ± 0.0019	0.9877 ± 0.0031	0.9613 ± 0.0058	0.9405 ± 0.0095
1:3	0.9927 ± 0.0013	0.9829 ± 0.0027	0.9650 ± 0.0042	0.9264 ± 0.0088
1:4	0.9934 ± 0.0016	0.9810 ± 0.0034	0.9696 ± 0.0022	0.9188 ± 0.0058

It should be noted that the positive-to-negative sample ratio analysis evaluates the robustness of GATROF under different pseudo-negative sampling settings, but it does not represent strict cold-start validation. In the current pair-level cross-validation protocol, drugs and microbes in the test pairs may still appear in the training folds through other association pairs. Therefore, the current results mainly reflect missing-link prediction among known drugs and microbes rather than prediction for completely unseen entities.

## Case study

To better illustrate the effectiveness of GATROF, we conducted case studies on two well-known drugs and one microbe. The first drug selected was ciprofloxacin, a synthetic second-generation quinolone antibacterial agent with broad-spectrum antibacterial activity and bactericidal efficacy, which can be used to treat diseases caused by influenza *bacillus*, *Escherichia coli*, and pneumococcus-specific polysaccharides (Xu et al., 2021). *In vitro* and *in vivo* studies of ciprofloxacin have reported a very low incidence of resistant microbes (Veličković et al., 2017).

In addition, Alhajj et al. (Köhler et al., 2008) developed a ciprofloxacin dry powder for inhalation therapy for pulmonary infection in cystic fibrosis. Golapudi et al. verified that ciprofloxacin suppresses TNF-α-induced HIV secretion from U1 cells (Hattori et al., 2010). Table 6 shows that 17 of the top 20 predicted candidate microbes associated with ciprofloxacin have been confirmed by published journal articles.

**TABLE 6:** Top 20 identified microbes associated with ciprofloxacin.

Microbe	Evidence	Microbe	Evidence
*Pseudomonas aeruginosa*	PMID: 37855639	*Staphylococcus* capitis	PMID: 28153968
Kocuria rhizophila	PMID: 32243151	*Enterobacter* ludwigii	PMID: 28223374
*Streptococcus* pyogenes	PMID: 33585043	*Vibrio* parahaemolyticus	PMID: 35727114
*Vibrio* anguillarum	PMID: 36735199	*Salmonella Typhi*	PMID: 32050286
Gardnerella vaginalis	PMID: 8109944	Hepatitis B virus F	PMID: 15365265
Coagulase-negative staphylococci	PMID: 35326828	Pseudoalteromonas sp	PMID: 31137680
Human herpesvirus 1	PMID: 25449284	Raoultella ornithinolytica	PMID: 32292483
Human respiratory syncytial virus B	Unconfirmed	*Streptococcus* parasanguinis	PMID: 21193474
*Serratia marcescens*	PMID: 2071875	*Candida* dubliniensis	Unconfirmed
Hafnia paralvei	Unconfirmed	*Bacteroides* vulgatus	PMID: 10795599

The first column lists the top 10 microbes, whereas the third column lists microbes ranked 11–20.

The second drug selected was moxifloxacin, a quinolone broad-spectrum antibacterial agent used to treat upper and lower respiratory tract infections, acute sinusitis, acute exacerbations of chronic bronchitis, community-acquired pneumonia, and skin and soft-tissue infections in adults (≥18 years). Januel C et al. (Kamneva, 2017) used moxifloxacin to treat hereditary spinal muscular atrophy (SMA). However, Inada K et al. (Cai et al., 2021) found that moxifloxacin induced aortic aneurysm and dissection in mice by increasing osteopontin.

Table 7 shows that 15 of the top 20 predicted candidate microbes have been confirmed by published journal articles to be associated with moxifloxacin, demonstrating the value of GATROF in clinical drug application and in the identification of potentially drug-associated microbes.

**TABLE 7:** Top 20 identified microbes associated with moxifloxacin.

Microbe	Evidence	Microbe	Evidence
*Streptococcus* pyogenes	PMID: 36853816	*Streptomyces* rubiginosus	Unconfirmed
*Bacteroides* vulgatus	PMID: 10795599	*Mycobacterium avium*	PMID: 31239192
*Staphylococcus* chromogenes	Unconfirmed	*Pseudomonas aeruginosa*	PMID: 33512346
Schistosoma	Unconfirmed	*Proteus* vulgaris	PMID: 12482994
*Pseudomonas* libaniensis	Unconfirmed	Dengue virus type 2	Unconfirmed
Human immunodeficiency virus 1	PMID: 34918028	Halomonas pacifica	Unconfirmed
*Staphylococcus* cohnii	PMID: 37998796	Burkholderia thailandensis	Unconfirmed
Trueperella pyogenes	PMID: 33552877	*Staphylococcus aureus*	PMID: 33936821
*Candida* dubliniensis	PMID: 30237975	Aggregatibacter actinomycetemcomitans	PMID: 26538521
Baker’s yeast	PMID: 25806673	Burkholderia pseudomallei	PMID: 21481571

The first column lists the top 10 microbes, whereas the third column lists microbes ranked 11–20.

The second microbe selected was *Escherichia coli*, a conditionally pathogenic bacterium that can, under certain conditions, cause gastrointestinal infection or various localized tissue and organ infections, such as urogenital infections in humans and many animals. Pathogenic *E. coli* causes more than 16.01 billion cases of dysentery and 1 million deaths every year, whereas nonpathogenic *E. coli* constitutes part of the normal intestinal microbiota of healthy mammals and birds. For example, the *E. coli* strain Nissle is expected to be used for the treatment of human diseases in addition to serving as a probiotic and therapeutic agent (Terp and Rybak, 1987). According to published journal articles, 15 of the top 20 predicted candidate drugs in Table 8 are associated with *E. coli*.

**TABLE 8:** Top 20 predicted drugs associated with *Escherichia coli*.

Drug	Evidence	Drug	Evidence
(10R,11R)-hydroxycarbene	PMID: 26273725	14-Alpha-lipoyl andrographolide	PMID: 19652378
3,5-Diiodotyrosine	PMID: 36323433	2-(4,5-Dibromo-1-methyl-1H-pyrrol-2-yl)-5-(2,4-dichlorophenyl)-1,3,4-oxadiazole	Unconfirmed
Cefotiam	PMID: 32828676	3-[(Prop-2-ene-1-sulfinyl)sulfanyl]prop-1-ene	Unconfirmed
C4-HSL	PMID: 24269673	3,5-Dimethyl benzyl dodecyl beta-maltoside	Unconfirmed
para-Benzoquinone	PMID: 27134027	Magainin-I	PMID: 30277857
Hinokitiol	PMID: 17927050	(1E)-1-{[(1E)-prop-1-ene-1-sulfinyl]sulfanyl}prop-1-ene	Unconfirmed
Hexameric peptide	PMID: 26251445	Dicyclohexylamine	PMID: 6508744
para-ethylaniline	PMID: 28383815	(10R,11R)-hydnocarpin D	PMID: 26273725
3-{2-[(1S,2R,4aR,8aR)-1,2,4a,5-tetramethyl-1,2,3,4,4a,7,8,8a-octahydronaphthalen-1-yl]ethyl}-5-methylidene-N-phenyl-2,5-dihydrofuran-2-amine	Unconfirmed	3,4-Dichloro-cinnamaldehyde	PMID: 27939874
hLF1-11	PMID: 24631659	Paromomycin	PMID: 60235

The first column lists the top 10 drugs, whereas the third column lists drugs ranked 11–20.

Overall, these case studies provide qualitative biological support for the predictions generated by GATROF. The top-ranked candidates are mostly related to known antimicrobial activity, microbial pathogenicity, infection-related diseases, or reported drug–microbe responses. This observation is consistent with the design of GATROF, which integrates heterogeneous biological information, topological similarity, and learned graph representations to prioritize biologically plausible drug–microbe associations. For candidates marked as unconfirmed, we conservatively regard them as associations not yet reported in the queried literature rather than definitive false positives.

## Conclusion and discussion

To identify potential drug–microbe associations, we developed an integrated predictive framework named GATROF by combining GAT with Rotation Forest. Comparative experiments and case studies demonstrated that GATROF achieved satisfactory predictive performance in identifying potential drug–microbe associations. The main contribution of GATROF lies in the task-oriented integration of heterogeneous biological information, graph attention-based topological representation learning, and Rotation Forest classification for sparse drug–microbe association prediction. This framework may also be extended to other biological-entity association prediction tasks with similar heterogeneous network structures. The ablation results indicate that the predictive power of GATROF is mainly supported by association profiles and biological similarity features, whereas GAT-derived topological representations provide complementary graph-based information within the heterogeneous feature fusion framework. Nevertheless, GATROF still requires further improvement. For example, more biological data, such as microbial sequencing information, could be added to the feature-selection component (Barman Balf et al., 2000). In addition, because the available microbe–drug association data are still sparse, the model may suffer from overfitting or limited generalization in some prediction scenarios. To address this problem, data augmentation may also be considered. Because the database is not updated in real time, which may affect the practical application of the model may be affected. We may therefore consider reconstructing a more comprehensive database (Xiang et al., 2020).

Another limitation is that the current validation mainly follows pair-level cross-validation on the MDAD benchmark dataset. Although the positive pairs in each test fold were excluded from model training, the same drugs or microbes may still appear in both training and test folds through other association pairs. Therefore, this setting mainly evaluates missing-link prediction among known entities and does not fully reflect the difficulty of practical cold-start discovery scenarios involving completely unseen drugs or microbes. More stringent validation settings, including unseen-drug prediction, unseen-microbe prediction, double-cold-start prediction, and independent external validation, will be important directions for future work to further evaluate the practical generalizability of GATROF.

## Data Availability

The original contributions presented in the study are included in the article/Supplementary Material, further inquiries can be directed to the corresponding authors.

## References

[B1] Andersen P. I. Ianevski A. Lysvand H. Vitkauskiene A. Oksenych V. Bjørås M. (2020). Discovery and development of safe-in-man broad-spectrum antiviral agents. Int. J. Infect. Dis. 93, 268–276. 10.1016/j.ijid.2020.02.018 32081774 PMC7128205

[B2] Barman Balfour J. A. Lamb H. M. (2000). Moxifloxacin: a review of its clinical potential in the management of community-acquired respiratory tract infections. Drugs 59, 115–139. 10.2165/00003495-200059010-00010 10718103

[B3] Cai L. Lu C. Xu J. Meng Y. Wang P. Fu X. (2021). Drug repositioning based on the heterogeneous information fusion graph convolutional network. Brief. Bioinform 22 (6), bbab319. 10.1093/bib/bbab319 34378011

[B4] Chen Y. Li H. Wang L. (2025). Deep homo-heterogeneous association mining with hybrid scholars and multidimensional mixed moment networks: embedding-driven prediction of microbe-drug interactions. Comput. Biol. Med. 196 (Part A), 110694. 10.1016/j.compbiomed.2025.110694 40644894

[B5] Cheng X. Qu J. Song S. Bian Z. (2022). Neighborhood-based inference and restricted boltzmann machine for microbe and drug associations prediction. PeerJ 10, e13848. 10.7717/peerj.13848 35990901 PMC9387521

[B6] Deng L. Huang Y. Liu X. Liu H. (2022). Graph2MDA: a multi-modal variational graph embedding model for predicting microbe-drug associations. Bioinformatics 38 (4), 1118–1125. 10.1093/bioinformatics/btab792 34864873

[B7] Hattori M. Tanaka N. Kanehisa M. Goto S. (2010). SIMCOMP/SUBCOMP: chemical structure search servers for network analyses. Nucleic Acids Res. 38 (Suppl. 2), W652–W656. 10.1093/nar/gkq367 20460463 PMC2896122

[B8] Huang H. Sun Y. Lan M. Zhang H. Xie G. (2023). GNAEMDA: microbe-drug associations prediction on graph normalized convolutional network. IEEE J. Biomed. Health Inf. 27, 1635–1643. 10.1109/JBHI.2022.3233711 37022036

[B9] Human Microbiome Project Consortium (2012). Structure, function and diversity of the healthy human microbiome. Nature 486 (7402), 207–214. 10.1038/nature11234 22699609 PMC3564958

[B10] Kamneva O. K. (2017). Genome composition and phylogeny of microbes predict their co-occurrence in the environment. PLoS Comput. Biol. 13 (2), e1005366. 10.1371/journal.pcbi.1005366 28152007 PMC5313232

[B11] Köhler S. Bauer S. Horn D. Robinson P. N. (2008). Walking the interactome for prioritization of candidate disease genes. Am. J. Hum. Genet. 82 (4), 949–958. 10.1016/j.ajhg.2008.02.013 18371930 PMC2427257

[B12] Kuang H. Zhang Z. Zeng B. Liu X. Zuo H. Xu X. (2024). A novel microbe-drug association prediction model based on graph attention networks and bilayer random forest. BMC Bioinforma. 25, 78. 10.1186/s12859-024-05687-9 38378437 PMC10877932

[B13] Li H. Hou Z. J. Zhang W. G. Qu J. Yao H. B. Chen Y. (2023). Prediction of potential drug-microbe associations based on matrix factorization and a three-layer heterogeneous network. Comput. Biol. Chem. 104, 107857. 10.1016/j.compbiolchem.2023.107857 37018909

[B14] Long Y. Luo J. (2021). Association mining to identify microbe drug interactions based on heterogeneous network embedding representation. IEEE J. Biomed. Health Inf. 25 (1), 266–275. 10.1109/JBHI.2020.2998906 32750918

[B15] Long Y. Wu M. Kwoh C. K. Luo J. Li X. (2020). Predicting human microbe–drug associations via graph convolutional network with conditional random feld. Bioinformatics 36 (19), 4918–4927. 10.1093/bioinformatics/btaa598 32597948 PMC7559035

[B16] Ma Q. Tan Y. Wang L. (2023). GAHNNMDA: a computational model for predicting potential human microbe-drug associations based on graph attention network and HNN-based classifier. BMC Bioinforma. 24, 35. 10.1186/s12859-023-05158-7 36732704 PMC9893988

[B17] Qu J. Song Z. Cheng X. Jiang Z. Zhou J. (2023). A new integrated framework for the identification of potential virus-drug associations. Front. Microbiol. 14, 1179414. 10.3389/fmicb.2023.1179414 37675432 PMC10478006

[B18] Rajput A. Thakur A. Sharma S. Kumar M. (2018). aBiofilm: a resource of anti-biofilm agents and their potential implications in targeting antibiotic drug resistance. Nucleic Acids Res. 46 (D1), D894–D900. 10.1093/nar/gkx1157 29156005 PMC5753393

[B19] Rodriguez J. J. Kuncheva L. I. Alonso C. J. (2006). Rotation forest: a new classifer ensemble method. IEEE Trans. Pattern Analysis Mach. Intell. 28, 1619–1630. 10.1109/TPAMI.2006.211 16986543

[B20] Say N. Tanvir F. Keita M. Chebbah L. Islam M. I. K. Akbas E. (2025). MCL-DMD: multi-modal contrastive learning for drug–microbe–disease association prediction. BCB 52, 1–6. 10.1145/3765612.3767307

[B21] Sun Y. Z. Zhang D. H. Cai S. B. Ming Z. Li J. Q. Chen X. (2018). MDAD: a special resource for microbe-drug associations. Front. Cell Infect. Microbiol. 8, 424. 10.3389/fcimb.2018.00424 30581775 PMC6292923

[B22] Tanvir F. Saifuddin K. M. Hossain T. Bagavathi A. Akbas E. (2024). HeTAN: heterogeneous graph triplet attention network for drug repurposing. DSAA, 1–10. 10.1109/DSAA61799.2024.10722832

[B23] Terp D. K. Rybak M. J. (1987). Ciprofoxacin. Drug Intell. Clin. Pharm. 21, 568–574. 10.1177/1060028087021007-801 3301247

[B24] Thiele I. Heinken A. Fleming R. M. (2013). A systems biology approach to studying the role of microbes in human health. Curr. Opin. Biotechnol. 24 (1), 4–12. 10.1016/j.copbio.2012.10.001 23102866

[B25] Veličković P. Cucurull G. Casanova A. Romero A. Liò P. Bengio Y. (2017). Graph Attention Networks. arXiv:1710.10903. 10903. 10.48550/arXiv.1710.10903

[B26] Wang L. Tan Y. Yang X. Kuang L. Ping P. (2022). Review on predicting pairwise relationships between human microbes, drugs and diseases: from biological data to computational models. Briefngs Bioinform 23 (3), bbac080. 10.1093/bib/bbac080 35325024

[B27] Xiang Y.-T. Li W. Zhang Q. Jin Y. Rao W. W. Zeng L. N. (2020). Timely research papers about COVID-19 in China. Lancet 395 (10225), 684–685. 10.1016/S0140-6736(20)30375-5 32078803 PMC7133590

[B28] Xu D. Xu H. Zhang Y. Wang M. Chen W. Gao R. (2021). MDAKRLS: predicting human microbe–disease association based on Kronecker regularized least squares and similarities. J. Transl. Med. 19 (1), 1–12. 10.1186/s12967-021-02732-6 33579301 PMC7881563

[B29] Xuan P. Gao L. Sheng N. Zhang T. Nakaguchi T. (2020). Graph convolutional autoencoder and fully-connected autoencoder with attention mechanism based method for predicting drug–disease associations. IEEE J. Biomed. Health Inf. 25 (5), 1793–1804. 10.1109/JBHI.2020.3039502 33216722

[B30] Yu Ge Chen F. Chen H. Tang S. Liang M. Liu X. (2026). BRMDA: prediction model for potential microbe-drug associations based on bilinear attention networks and random forest. Front. Genet. 17, 1757318. 10.3389/fgene.2026.1757318 41960144 PMC13061380

[B31] Zhu L. Duan G. Yan C. Wang J. (2019). “Prediction of microbe-drug associations based on KATZ measure,” in 2019 IEEE International Conference on Bioinformatics and Biomedicine (BIBM). San Diego, CA, USA, 183–187. 10.1109/BIBM47256.2019.8983209

[B32] Zhu L. Wang J. Li G. Hu X. Ge B. Zhang B. (2021). Predicting microbe–drug association based on similarity and semi-supervised learning. Am. J. Biochem. Biotechnol. 17 (1), 50–58. 10.3844/ajbbsp.2021.50.58

